# A folate/RGD‐dual‐functionalized mesoporous silica nanoparticles targeting GABA‐p38 MAPK‐MRTFs/SRF signaling pathway in rheumatoid arthritis

**DOI:** 10.1002/ctm2.408

**Published:** 2021-05-07

**Authors:** Xinqi Deng, Ke Hou, Lei Yang, Zhengju Zhang, Wen Gu, Xiangwei Bu, Hui Liu, Weiguo Ma, Kang Wang, Hua Bai, Honghong Zhang, Dali Wang, Chunguo Wang, Huiyuan Zhang, Fengxian Meng

**Affiliations:** ^1^ School of Life Science Beijing University of Chinese Medicine Beijing China; ^2^ CAS Key Laboratory of Nanosystem and Hierarchical Fabrication CAS Center for Excellence in Nanoscience National Center for Nanoscience and Technology Beijing China; ^3^ Department of nephropathy The Third Affiliated Hospital of Beijing University of Chinese Medicine Beijing China; ^4^ Dongfang Hospital of Beijing, University of Traditional Chinese Medicine Beijing China; ^5^ Beijing Hospital of Traditional Chinese Medicine Capital Medical University Beijing China; ^6^ Department of Traditional Chinese Medicine School of Medicine Xiamen University Xiamen China; ^7^ Department of acupuncture Guangwai Hospital Beijing China; ^8^ Department of rheumatology Shunyi Hospital, Beijing Traditional Chinese Medicine Hospital Beijing China; ^9^ Community Health Service Beijing China; ^10^ Beijing Research Institute of Chinese Medicine Beijing University of Chinese Medicine Beijing China; ^11^ Center for Immunology and Hematology, State Key Laboratory of Biotherapy West China Hospital Sichuan University Chengdu China

Dear Editor,

Rheumatoid arthritis (RA) is an autoimmune disease that is associated with burdened personal, social, and economic costs. Enhanced understanding of RA pathogenesis and the development of effective therapies is pressingly needed. Seeking effective solution for diseases from natural medicine has become an attractive point of providing a new perspective for drug development. Polydatin (PD) has been found to be beneficial to arthritis. However, the pharmacological action of PD is weak in clinical practice due to poor aqueous solubility, chemical instability in aqueous alkaline medium, and extensive first‐pass metabolism. Biodegradable nanoparticle, which is of better solubility and lower degradation, could be a good choice for PD targeted delivery to overcome these obstacles.

With advantages in straightforward synthesis,^1^, ^2^, ^3^ mesoporous silica nanoparticles (MSNs) were chosen for developing an efficient and safe nanocarrier for PD in this study. We developed PD@MSN‐FA/RGD (Figure 1A, Figures S1–S8, Table S1) as a dual‐target nano‐drug delivery system that aimed at folate receptor and ανβ3 integrin receptor. Drug release profiles of PD@MSN‐FA/RGD were illustrated to be sustained and mass ratio dependent (Figure 1B). To further evaluate the possibility of in vivo application of PD@MSN‐FA/RGD, the hemolysis test was performed in vitro and in vivo. No significant difference was observed between MSN and control (‐) samples. It was demonstrated that no false negative or false‐positive results, which could be caused by adsorption of hemoglobin on particle surfaces^4^ or toxicity of the residual surfactant,^5^ occurred in hemolysis assay of PD@MSN‐FA/RGD. No significant hemolysis occurred with the concentration below 13.80 mg/ml, and excellent biocompatibility was displayed with the concentration below 4.60 mg/ml in vitro (Figure 1E and F). Likewise, no hemolysis effect of PD@MSN‐FA/RGD was observed with dosage below 438.28 mg/kg in mice (Table S2). Moreover, no significant acute toxicity was observed in mice subjected to PD@MSN‐FA/RGD administration (Table S3, Figure S9) except for slight or local liver lesions, including diffuse swelling (Figure S9B5) and local necrosis (Figure S9B3 and B4) in some samples. How these lesions being induced deserved further investigation. In addition, the performance of PD@MSN‐FA/RGD in vivo was illustrated by employing a selective ion monitoring model in UHPLC‐Q‐Exactive Orbitrap MS. It displayed an extremely low content of PD@MSN‐FA/RGD in tissues except plasma, synovial fluid, and synovial membrane, suggesting a significantly targeted delivery of PD@MSN‐FA/RGD injection comparing with that of polydatin injection (Figure 1C and D, Figure S10). No significant degradation product was detected in the liver upon PD@MSN‐FA/RGD injection, suggesting a lower degradation of PD with nanoparticles embedding (Figure S11).

**FIGURE 1:**
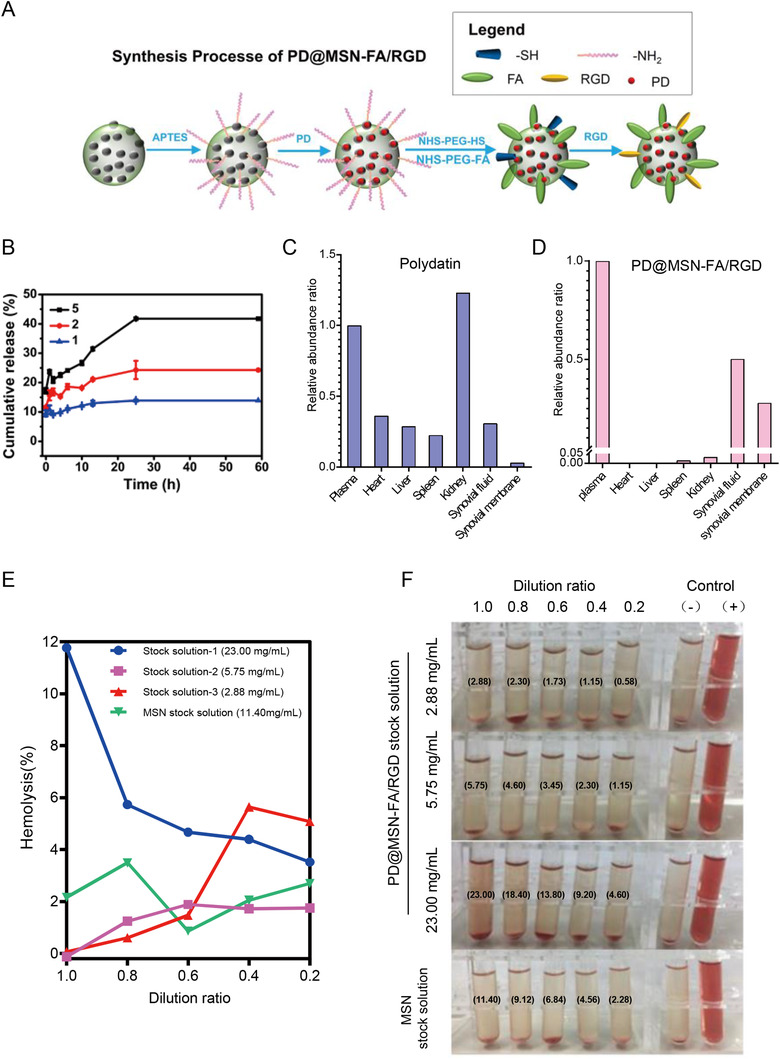
(**A)** The synthesis process of PD@MSN‐FA/RGD. (**B)** Drug release profiles of PD@MSN‐FA/RGD under different mass ratio (MSN/PD) of 5, 2, and 1. (**C and D)** Relative abundance of polydatin in plasma, heart, liver, spleen, kidney, synovial fluid, and synovial membrane tissue of rats subjected to polydatin injection **(C)** or PD@MSN‐FA/RGD injection **(D)**. (**E)** Hemolysis assay results of MSNs as well as PD@MSN‐FA/RGD at multiple concentrations. (**F)** Photographs of RBCs treated with PD@MSN‐FA/RGD or MSNs under different conditions. (‐) and (+) controls are the RBCs in 0.9% sodium chloride or water, respectively. Final concentrations (mg/ml) were marked on the tubes

In the collagen‐induced arthritis (CIA) model, joint damages were observed significantly improved upon PD@MSN‐FA/RGD treatment based on vertical and horizontal diameters of hind legs and ankle joints, the thickness of footpads, as well as histopathology changes in synovial membrane and cartilage tissue (Figure 2, Tables S4–S10).

**FIGURE 2:**
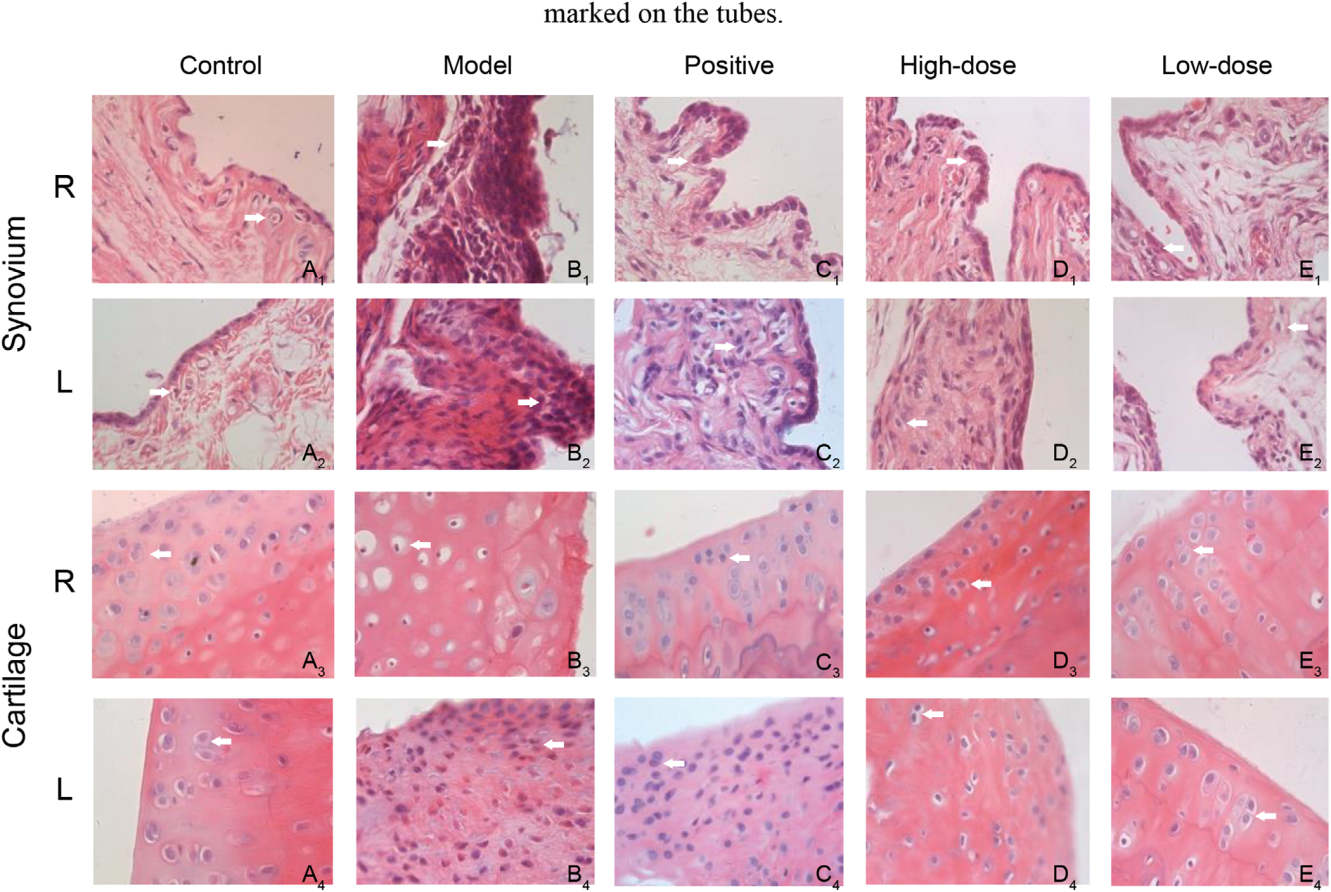
H&E staining of pathological changes in ankle joints responding to different treatments. Images of right‐synovium were shown in **A_1_, B_1_, C_1_, D_1_**, and **E_1_**; left‐synovium were shown in **A_2_, B_2_, C_2_, D_2_**, and **E_2_;** right‐cartilage were shown in **A_3_, B_3_, C_3_, D_3_,** and **E_3_**; left‐cartilage were shown in **A_4_, B_4_, C_4_, D_4_,** and **E_4_**

The metabolic profile of PD@MSN‐FA/RGD in synovial fluid was further studied. PCA and OPLS‐DA results showed the availability of CIA modeling as well as the significant influence of PD@MSN‐FA/RGD (R2X = 0.62, R2Y > 0.6, Q2 > 0.6) (Figure S12). Note that 115 molecules were identified from 187 ions that contributed to good separations among groups (VIP > 1 in ESI+, *p* < 0.05). Among the metabolites identified, 26 were found upregulated and 2 were downregulated responding to CIA modeling and significantly reversed by positive drug methotrexate and PD@MSN‐FA/RGD treatment (Table S11, Figure S13).

Moreover, metabolomics analysis was performed by employing IPA software. High‐dose and low‐dose PD@MSN‐FA/RGD treatment showed compatible metabolism on the CIA model (Figure 3A and B). Numerous metabolic pathways shifted toward rheumatoid arthritis‐related intermediates and metabolic endpoints were suggested by pathway analysis (Figure 3C and D). Moreover, dramatical downregulation of PD@MSN‐FA/RGD on D‐serine, guanine, and hypoxanthine (Figure 3E–G) indicated a strong inhibition of fibroblasts proliferation. With changes in guanine, d‐serine, l‐arginine, choline, kynurenic acid, and 5‐methylcytosine, it was suggested that mitogen‐activated protein kinase (MAPK) family proteins playing key roles in PD@MSN‐FA/RGD regulation (Figure 3I). Increased expression of p38 MAPK, a crucial molecule in the progression of RA,^6^ was found significantly inversed upon administration of PD@MSN‐FA/RGD in synovial tissues (Figure 3L, P, and S). Moreover, we found δ‐guanidinovaleric acid (Figure 3H), aγ‐aminobutyric acid (GABA)‐receptor antagonist, increased significantly responding to CIA modeling, suggesting inhibition of GABA receptor. These changes were reversed by PD@MSN‐FA/RGD administration, suggesting the promotion on GABA signaling. Based on the effect of inhibited p38 MAPK on joint inflammation, and the inhibitory role of GABA in p38 MAPK signaling, a hypothesis^7^ was proposed that GABA may downregulate p38 MAPK activity to suppress inflammation in RA.

**FIGURE 3:**
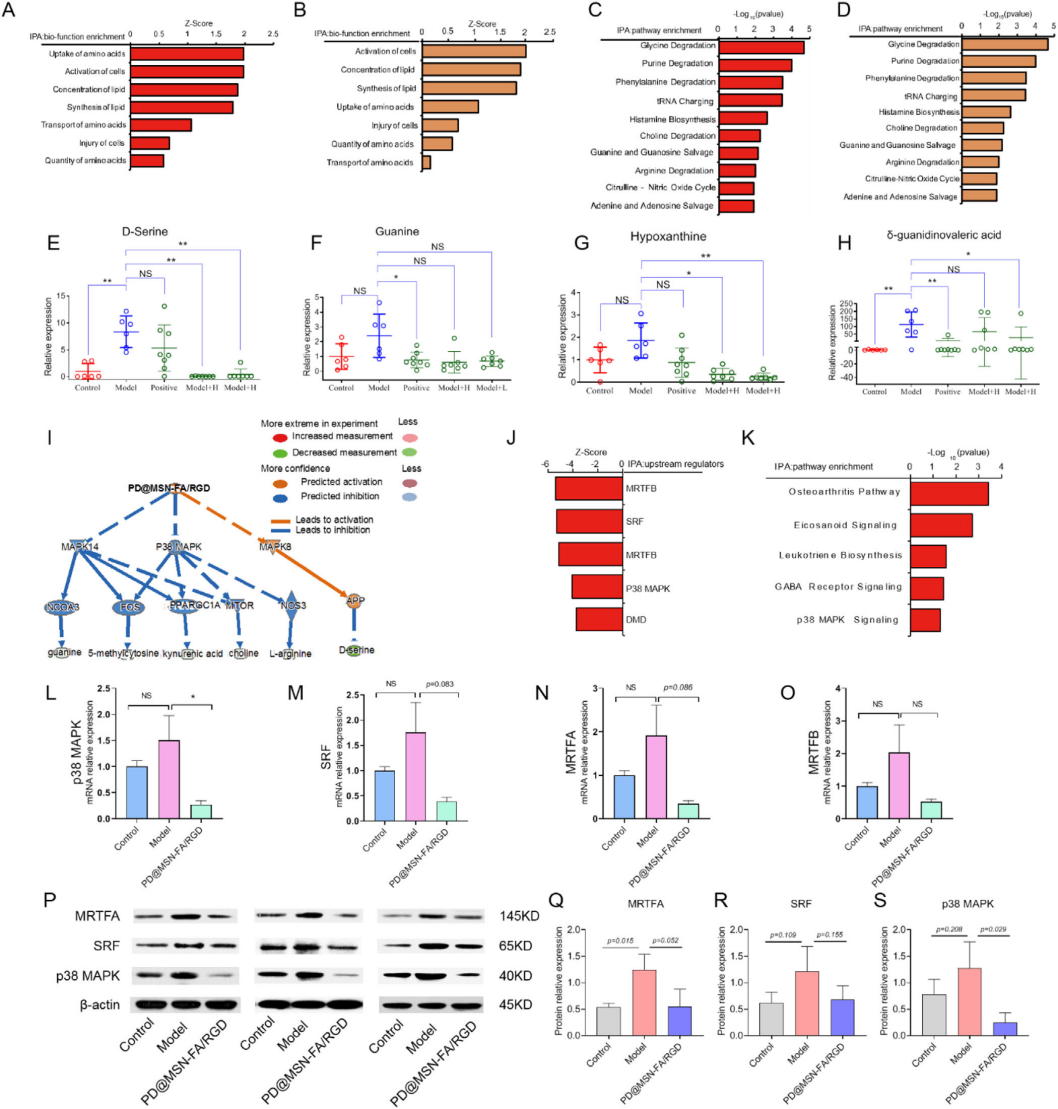
Metabolomic and transcriptomic studies on PD@MSN‐FA/RGD regulations in RA. (**A–D)** Biological function and pathway analysis enriched with IPA software based on metabolome data. Results based on data of model group versus high‐dose PD@MSN‐FA/RGD group **(A, C)**; results based on data of model group versus low‐dose PD@MSN‐FA/RGD group **(B, D)**; (**E–H)** production changes of d‐serine **(E)**, guanine **(F)**, hypoxanthine **(G)**, and δ‐guanidinovaleric acid **(H)**; (**I)** key proteins in PD@MSN‐FA/RGD regulation predicted by IPA; (**J–K)** top pathways **(J)** and up‐stream proteins **(K)** predicted with IPA software based on transcriptome data; (**L–O)** mRNA expression changes of p38 MAPK **(L)**, SRF **(M)**, MRTFA **(N)**, and MRTFB **(O)**; **(P–S)** protein expression changes **(P)** of MRTFA **(Q)**, SRF **(R)**, and p38 MAPK **(S)**. Data are presented as mean ± SEM, *t‐test*, *n* = 6, *p < 0.05, **p < 0.01

How does PD@MSN‐FA/RGD suppress fibroblasts proliferation via the GABA‐p38 MAPK pathway? To address this question, a transcriptomic study was carried out. Pathway analysis showed that GABA‐p38 MAPK signaling is significantly regulated by PD@MSN‐FA/RGD, consistent with that of metabolome study (Figure 3J). Intriguingly, myocardin‐related transcription factor A (MRTFA), myocardin‐related transcription factor B (MRTFB), and serum response factor (SRF) that played key roles in fibroblast activation were predicted as potential upstream regulators in PD@MSN‐FA/RGD's regulation (Figure 3K). Upregulated mRNA expressions of MRTFA, MRTFB, and SRF in the CIA model were found reversed with PD@MSN‐FA/RGD in synovial tissues to different extents (Figure 3L–O). Similar regulations were also observed at the protein level (Figure 3P–S). p38 MAPK cascades could promote the formation of ternary nucleoprotein complex and activate the early response gene transcription initiated by SRF.^8^ SRF functions in partnership with MRTFA/B and acts as a key mediator in fibroblasts activation.^9^, ^10^ Taken together, it was suggested that PD@MSN‐FA/RGD alleviated inflammation and fibroblasts proliferation in RA by regulating GABA‐p38 MAPK‐MRTFs/SRF signaling pathway (Figure 4).

**FIGURE 4:**
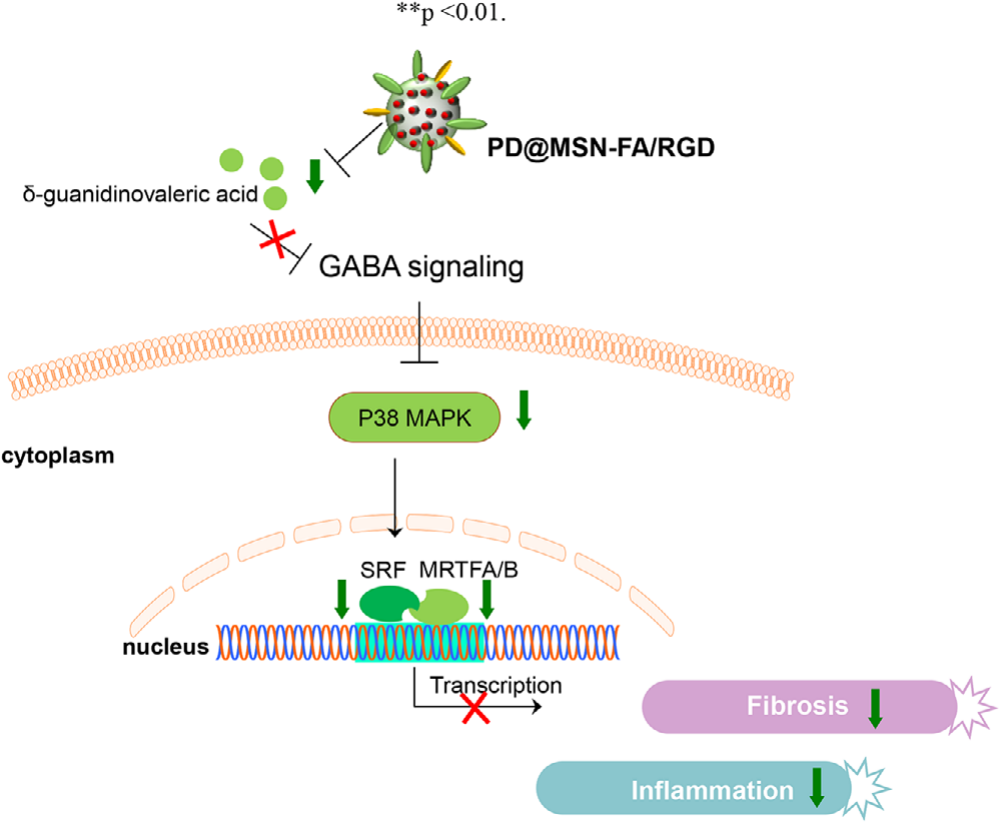
The regulatory mechanism of PD@MSN‐FA/RGD in vivo

In this work, a folate/RGD‐dual‐functionalized mesoporous silica nanoparticles (MSNs‐FA/RGD) with reproducible and stable production, as well as good biocompatibility, was developed to carry PD to the synovial area to achieve promising clinical translation and application in RA therapy. Protective effects of PD@MSNs‐FA/RGD on rheumatoid arthritis were illustrated with CIA modeling rats. PD@MSNs‐FA/RGD was found targeting on GABA‐p38 MAPK‐MRTFs/SRF signaling pathway in vivo to exert its beneficial influence. We reported a novel drug of RA with both characterization and regulatory mechanism carefully discussed and clarified.

## CONFLICT OF INTEREST

The authors have declared no conflict of interest.

## Supporting information



Supplementary Information

Figure S1. (A and B) TEM images of MSNs, and it was observed that MSNs are spherical nanoparticles with a diameter of about 80 nm.
Figure S2. Hydrodynamic diameter (A) and Zeta potential (B) of MSNs. The average hydrodynamic size of MSNs is 277.5 nm and their surface charge is 28.9 mV.
Figure S3. SEM (A) and TEM (B) images of MSNs. SEM (C) and TEM (D) images of MSN‐NH_2_. No significant influence of ‐NH_2_ modification on the spherical morphology, size, as well as mesoporous property of MSNs was observed.
Figure S4. Zeta potential of MSN‐NH_2_. The surface charge is about 16.9 mV.
Figure S5. The FTIR spectra of MSNs and MSN‐NH_2_. A new FTIR spectra band appeared at 3421 cm‐1 after modification due to the stretching vibration of ‐NH_2_ groups.
Figure S6. The linear relationship between the concentration of PD and their optical absorption intensity. The fitted linear equation is y = 0.04009 + 0.02998x, R^2^ = 0.99897.
Figure S7. (A) N_2_ adsorption‐desorption isotherms and (B) pore‐size distributions of MSNs. (C) N_2_ adsorption‐desorption isotherms and (D) pore‐size distributions of PD@MSNs.
Figure S8. (A) UV‐Vis spectra of PD@MSN‐NH_2_, NHS‐PEG‐FA, PD@MSN‐FA/SH. (B) Zeta potential of PD@MSN‐NH_2_, PD@NHS‐FA/SH, PD@MSN‐FA/RGD.
Figure S9. H&E staining of pathological changes in response to different dosages of PD@MSN‐FA/RGD after 14 days’ administration.
Figure S10. UPLC‐Q exactive quantitative analysis of polydatin in plasma, heart, liver, spleen, kidney, synovial fluid, and synovial membrane tissue of rats which subject to PLN injection based on selective ion monitoring (SIM)‐based method.
Figure S11. Metabolic trait of polydatin in vivo. 7 major metabolites generated from polydatin degradation were detected in the liver of rats which subject to polydatin injection, while none significant degradation product was detected upon PD@MSN‐FA/RGD injection.
Figure S12. Multi‐dimensional statistics of metabolomics data. A–B. PCA analysis based on LC‐MS data obtained from synovial fluid of Control, Model, Positive, high‐dose PLN, and low‐dose PLN group. (A) Result based on HILIC mode data; (B) Result based on RP‐C18 mode data; C‐H. OPLS‐DA on LC‐MS data of common metabolites of the control group, model group, positive group, high‐dose PLN group, and low‐dose PLN group. (C, F) Control vs. Model groups under HILIC and RP‐C_18_ mode. (D, G) Model vs. high‐dose PLN groups under HILIC and RP‐C_18_ mode (E, H) Model vs. low‐dose PLN groups under HILIC and RP‐C_18_ mode; I‐N. Robustness assessments of OPLS‐DA model.
Figure S13. Hierarchical clustering analysis on common differential metabolites detected and identified in each group.
Table S1. The loading content and entrapment efficiency of PD@MSN and PD@MSN‐NH_2_.
Table S2. OD values of serum in mice subjecting to different dosages of PD@MSN‐FA/RGD.
Table S3. Organ coefficients of heart, liver, spleen, lung, and kidney in mice subjecting to different dosages of PD@MSN‐FA/RGD.
Table S4. Horizontal diameters of right hind legs (mm)
Table S5. Vertical diameters of right hind legs (mm)
Table S6. Horizontal diameters of left hind legs (mm)
Table S7. Vertical diameters of left hind legs (mm)
Table S8. Thickness of right hind footpads (mm)
Table S9. Thickness of left hind footpads (mm)
Table S10. Vertical and horizontal diameters of left/right ankle joints
Table S11. Differential metabolites being detected and identified.
