# Supplementary material for: A folate/RGD‐dual‐functionalized mesoporous silica nanoparticles targeting GABA‐p38 MAPK‐MRTFs/SRF signaling pathway in rheumatoid arthritis
Source: Clin Transl Med. 2021 May 7;11(5):e408. doi: 10.1002/ctm2.408 (PMC8103722; doi:10.1002/ctm2.408)
Supplement: Supplementary file 1 — Supplementary Information [file CTM2-11-e408-s001.docx]

**Supplementary information**

**Reagents and drugs**

Tetraethylorthosilicate (TEOS), hexadecyltrimethylammonium chloride (CTAC), triethanolamine (TEA), dimethylacetamide (DMAC), aminopropyltriethoxysilane (APTES), Ethylenediaminetetraacetic acid disodium salt (EDTA), N-hydroxylsuccinimide-polyethylene glycol- folic acid (NHS-PEG-FA, MW 2000) and N-hydroxylsuccinimide-polyethylene glycol-thiol (NHS-PEG-SH, MW 2000) were obtained from Shanghai ZZbio Co., ltd. The monomaleimide-functionalized RGD peptide (Mal-RGD) was bought from GL Biochem (Shanghai) Ltd. Polydatin (CAS: 27208-80-6, H-012-181216. Chengdu Herbpurify CO., LTD., China); Type Ⅱ collagen (CⅡ, Lot No. 180390, Chondrex Co., Ltd., USA.); Complete Freund's adjuvant (CFA, Lot No. SLBV0593, Sigma-Aldrich Co., Ltd., USA.); Water for injection (WFI, Lot No. 1708508, Sinopharma Rongsheng Co., Ltd., China); Aldehyde (Lot No. F20180824, Sinopharma chemical reagent Co., Ltd., China); Acetonitrile, methanol and formic acid (HPLC grade, Fisher Co., Ltd., MA); Methotrexate injection (Lot No.T87971, Pfizer Co., Ltd., USA.).

**PD@MSNs-FA/RGD preparation**

*Synthesis of MSNs*

The MSNs were synthesized according to previous procedures with modification.[^1^](#_ENREF_1) Briefly, 10.0 g of CTAC was dissolved in 100 mL water. Then 250 μL of TEA was added into the above solution and kept stirred 1 h at 95°C. Following, 7.5 mL of the TEOS was dropwise added in the mixed solution and then stirred for another 1 h. The products were collected by centrifugation and then refluxed at 80°C in HCl/methanol for 6 h to remove the template CTAC. The MSNs were then collected by centrifugation and vacuum drying.

*Synthesis of MSNs-NH_2_*

1.0 g of the MSNs was added to 50 mL of toluene. Under nitrogen protection, 1.0 mL of APTES was dropwise added in the mixed solution and then refluxed for 24 h. The mixture was then extensively washed with toluene and ethanol for three times. Finally, the amine-functionalized MSNs were obtained after vacuum drying.

*Synthesis of PD@MSN-NH_2_*

The PD@MSNs were prepared by an impregnation method. Typically, 300 mg MSNs-NH_2_ was dissolved in 270 mL of ethanol, then 300 mg PD that dissolved in 30 mL of ethanol was added in the solution. The mixed solution was stirred for about 36 h at room temperature. The precipitate was centrifuged at 10000 rpm for 20 min and washed extensively with PBS to remove the redundant PD that on the surface of MSNs-NH_2_. Subsequently, PD@MSN-NH_2_ was obtained after vacuum drying. *Synthesis of PD@MSN-FA/RGD*

FA was firstly introduced to the PD@MSN-NH_2_ with the help of amido bond. Briefly, 10 mg NHS-PEG-FA and 10 mg NHS-PEG-SH were dissolved in 100 μL of DMAC. 10 mg of PD@MSN-NH_2_ that dissolved in 10 mL PBS solution (pH 7.4) was added to the above DMAC solution. After reacting for 4 h, the mixture was centrifuged at 10000 rpm for 20 min and washed extensively with PBS solution. Subsequently, the resultant product PD@MSN-FA/SH was collected by centrifugation and vacuum drying. The RGD peptide was further immobilized on the surface of PD@MSN-FA/SH via thiol‐maleimide addition. Typically, 5 mg Mal-RGD was dissolved in 5 mL PBS solution (pH 7.4), then 14.61 mg EDTA and 10 mg PD@MSN-FA/SH were added sequentially. The mixed solution was stirred for 2 h at room temperature, and then the product PD@MSN-FA/RGD were collected by centrifugation and vacuum drying.

**Characterization**

Scanning electron microscope (SEM) characterizations were performed on a Hitachi S4800 at 6.0 kV. The mesoporous silica nanoparticles were directly subjected to SEM observation. Transmission electron microscopy (TEM) images were obtained by a Tecnai G2 F20 S-TWIN with an acceleration voltage of 200 kV. Hydrodynamic particle size and zeta potential of samples with a concentration of around 1 mg/mL were measured by a Zetasizer Nano ZS at room temperature. Fourier transform infrared (FTIR) spectra were obtained on a Perkin Elmer Spectrum One spectrometer by using the KBr disk method. UV-vis spectra were recorded on a Hitachi U-3900 spectrophotometer (Hitachi Corportion, Tokyo, Japan). Nitrogen adsorption measurement was carried on a Micromeritics ASAP 2420-4 instrument. Before the measurements, the samples were outgassed at 150 ℃ under vacuum for four hours. The specific surface areas were determined from the linear portion of the Brunnauer-Emmett-Teller (BET) plots. The pore size distribution was calculated from the adsorption branch of nitrogen isotherms using the Barrett-Joyner-Halenda (BJH) method.

**In vitro drug release of PD@MSN-FA/RGD**

*In vitro* drug release studies were carried out in PBS solution with pH values of 7.4. Briefly, 10 mg of PD@MSN-FA/RGD was dispersed into 4 mL deionized water, then the solution was shocked at 37℃ at a speed of 150 rpm. At given time intervals, 2 mL of sample was withdrawn and centrifugated. The precipitate was redisposed in the same volume of fresh PBS and added to the previous solution. The OD of the supernatant at 485 nm was measured. The experiment was repeated in triplicate. The cumulative drug release was calculated according to

$Drug release \left( \% \right)=\left( \frac{M_{t}}{M_{i}} \right)\times100\%$

Where *M_t_* is the total amount of PD released from PD@MSN-FA/RGD at time *t*, and *M_i_* is the amount of PD initially loaded into the PD@MSN-NH_2_.

**Hemolysis assay**

For hemolysis assay in vitro, fresh blood was obtained from an adult male rabbit. 30 mL of the whole blood sample was added to 300 mL 0.9% sodium chloride and then was centrifuged at 1200 rpm for 15 min to remove the blood plasma and the surface layer. After being washed three times with 0.9% sodium chloride, red blood cells (RBCs) were finally diluted to a suspension at a concentration of 2% with 0.9% sodium chloride.

Multiple concentrations of PD@MSN-FA/RGD were generated with total assay volume of 5ml. Positive and negative control samples were prepared by adding 2.5 mL of water and 0.9 % sodium chloride, respectively to 2.5 mL of RBCs solution. After incubation at 37℃ for 3 h and centrifugation for 5 min at 1200 rpm, 100 μL of supernatants were transferred to a 96-well plate. The OD at 540 nm was measured by a microplate reader with the OD at 450 nm as a reference. The hemolytic ratio of the RBCs was calculated using the following formula:

$hemolytic ratio\%=\frac{{OD}_{sample}-{OD}_{negative control}}{{OD}_{positive control}-{OD}_{negative control}}\times100\%$

**Animals**

All the animal experiments, animal use and care were approved by the Animal Care Committee of the XXX Hospital, XXX University, in accordance with institutional animal care and use committee guidelines.

For hemolysis assay and acute toxicity test in vivo, ICR mice (20g ±2g) were purchased from Beijing Vital River Laboratory Animal Technology Co., Ltd. (Beijing, China; certification number: SCXK (Jing): 2012-0001). The mice were housed in the clean level condition animal housing facilities (certification number SYXK (Jing) 2016-0038) of BUCM, at a temperature of 22±1℃, a humidity of 55±5%, and a 12h light/dark cycle with free access to tap water and chow. All the protocols were reviewed and approved by the Animal Care Committee of BUCM, China. The animals were allowed to acclimatize for 3 days while being fed with a standard diet and water ad libitum. Mice were randomly and equally divided into five groups subjecting to different dosages of PD@MSN-FA/RGD injections: 438.28 mg/kg, 219.145mg/kg, 109.57mg/kg, and 54.78mg/kg, 0mg/kg. The control group received 1 mL 0.9% sodium chloride injection. 14 days after once injection, mice were anesthetized with 10% chloral hydrate and samples were obtained. Serum, heart, liver, spleen, lung, and kidney samples were obtained.

Male Sprague Dawley rats (220g ±20g) were purchased from Beijing Vital River Laboratory Animal Technology Co., Ltd. (Beijing, China; certification number: SCXK (Jing): 2012-0001). The rats were housed in the clean level condition animal housing facilities (certification number SYXK (Jing) 2016-0038) of BUCM, at a temperature of 22±1℃, a humidity of 55±5%, and a 12h light/dark cycle with free access to tap water and chow. All the protocols were reviewed and approved by the Animal Care Committee of BUCM, China. The animals were allowed to acclimatize for a week while being fed with a standard diet and water ad libitum.

For distribution and degradation investigations, rats were randomly divided into three groups: control group, polydatin group, and PD@MSNs-FA/RGD group. Rats in polydatin group and PD@MSNs-FA/RGD group were subjected to polydatin injection at 69mg/kg and PD@MSNs-FA/RGD injection (containing 45% PD) at 153.4mg/kg respectively. The control group received 1 mL 0.9% sodium chloride injection. Rats were anesthetized with 10% chloral hydrate (3.5 ml/kg body weight) and samples were obtained 2hrs after injection.

For H&E staining, metabolome, tanscriptome, and rt-qPCR assay, rats were randomly divided into five groups: control group, model group, positive group, high-dose PD@MSNs-FA/RGD group, and low-dose PD@MSNs-FA/RGD group. Except for the control group, rats were subjected to CIA. The animals in the positive group were treated with weekly methotrexate injection (0.5mg/kg); the high-dose PD@MSNs-FA/RGD group and low-dose PD@MSNs-FA/RGD group were treated with PD@MSNs-FA/RGD injection at 76.7mg/kg and 38.35mg/kg on alternate days; the control and model groups received 0.9% sodium chloride injection everyday (5 mL/kg). Four weeks after the last administration, rats were anesthetized with 10% chloral hydrate (3.5 ml/kg body weight) and samples were obtained.

**CIA modeling**

CIA rat model was established referring to method mentioned by Trentham D E et al. and DD Brand et al. [^2-4^](#_ENREF_2) Rats which subjected to CIA were intracutaneous injection of 0.2ml modeling agent (emulsion of complete Freund's adjuvant and type II collagen, 1mg/mL) at 7^th^ and 17^th^ day after adaptive feeding. After successful modeling, vertical and horizontal diameters of hind legs as well as the thickness of foot pads of rats were measured.

**H&E staining**

Hematoxylin and eosin (H&E) staining on synovial membrane samples was conducted referring to the protocol reported previously[^5^](#_ENREF_5). Images were captured using Olympus BX53 microscope (Tokyo, Japan) and analyzed using Image-Pro Plus 6.0 to calculate the IOD value [synovial](javascript:;) [membrane](javascript:;) samples

**Synovial fluid pretreatment and UHPLC-Q-Exactive Orbitrap mass spectrum (MS) analysis**

Synovial fluid was collected from knee joints into tubes containing methanol/acetonitrile solution (3:1, v/v) and centrifuged for 14000g, 15 min at 4℃ after vortex mixing. The supernatant was obtained and sample preparation was carried out by protein precipitation with 50% acetonitrile. To validate the analytical methodology, pooled quality control (QC) samples were prepared by mixing sample from each group. Five QC samples were analyzed before sample sequencing, and during the analysis of the sample sequence, one QC sample was run after every five injections.[^6^](#_ENREF_6)^,^ [^7^](#_ENREF_7)^.^ Metabolomics analysis was performed on a Thermo Scientific Vanquish UHPLC coupled to a Q Exactive Orbitrap MS system equipped with an electrospray ionization source operating. The operating parameters were as follows: spray voltage, 3.5 KV; sheath gas pressure, 35 arb; auxiliary gas pressure, 10 arb; capillary temp, 300 °C; ion source temp, 350 °C; scan modes, MS (Full Scan, m/z100-1200) and data-dependent acquisition MS^2^ (resolution 17,500, normalized collision energy 35 eV, stepped normalized collision energy 30 and 40 eV) and scan range, *m/z* 80–1200. Taking polar and minor polar components into consideration, ACQUITY UHPLC BEH C_18_ column (1.7um 2.1mm*100mm) and ACQUITY UHPLC HILIC column (1.7um 2.1mm*100mm) were equipped for complete data collection and analyses. The mobile phases were combined with 0.1% formic acid in water (A) and 0.1% formic acid in acetonitrile (B).

UHPLC HILIC gradient conditions: 0-10 min, 8-30% (A)；10-12 min, 30 -30 % (A) ；12-13 min, 30-8% (A)；13-15 min, 8-8% (A).

UHPLC C18 gradient conditions: 0-10 min, 70-15% (A)；10-11 min, 15 -15 % (A) ；11-12 min, 70-7% (A)；12-15 min, 70-70% (A).

**Data processing and analysis**

The raw data analysis was processed using Sieve software package version 2.1 (Thermo Fisher Scientific Inc., San Jose, CA, USA). Before chemometric analysis, the data from each sample were normalized to the sum of the peak area [^8^](#_ENREF_8). Metabolic data with intensity>5e^4^ in synovial fluid were subjected to Unit Variance Scaling and Mean-Centered data processing. PCA and OPLS-DA analyses was carried out with SIMCA-P 14.0 (Umetrics). Robustness of OPLS-DA model was assessed by a 200-times permutation test. Differential metabolites identification used Compound Discoverer 2.0 for spectral matching, combined with Metlin and HMDB databases. On the basis of automatic mapping analysis result, manual analysis on ion fragments was performed for further quality control. The obtained differential components were analyzed by MeV (Multi Experiment Viewer, v4.8, TIGR) for hierarchical clustering analysis and K-mean clustering analysis. All experimental data were expressed as means± *SD*, and examined by Oneway ANOVA test in the SPSS version 16 software package to calculate the statistical significance. P-value <0.05 were considered significant. IPA software was applied for bio-functions and pathways enrichment analysis based on differential metabolites converged from HILIC separate mode.

**RT-qPCR assay**

RNA was extracted with TRIzol (Invitrogen) method and reverse transcribed using the TIANScript RT Kit (TIANGEN) according to the manufacturer’s instructions. qPCR was performed in triplicate on Applied Biosystems 7500 Real-Time PCR System (Thermo Fisher). SYBR FAST qPCR Kit Master Mix (2×) Universal (KAPA Biosystems) was used according to the manufacturer’s instructions, each PCR reaction had a final volume of 20 μ l and 1μ l of cDNA. The running conditions were 10min at 95 °C, and then subjected to 45 cycles of 10 s at 95 °C and 60 s at 59 °C followed by a final extension of 65°C for 10 minutes]. All reactions were performed in triplicate. ACTB was used as reference controls. Data were calculated with the formula for relative FC=2‐ΔΔCT. PCR primer sequences used are as follow: ACTB, GCACCATGAAGATCAAGATCATT (forward) and TAACAGTCCGCCTAGAAGCATT (reverse); P38, TGAAGCTAGAGCTGGCAGAAGA (forward) and CCTATGGCATACCAGATCACAAC (reverse); MRTFA, GTGGGAAATGGGTGTGGCTA (forward) and GTTGAGTCCTGAGTCATGGTGC (reverse); MRTFB, TTGGCACTTACCTGATTGTTTCT (forward) and ATGATCACAAGACAAGGACGGA (reverse); SRF, ATGTTACCGAGCCAAGCTGGG (forward) and TGAATCAGCGCCTTGCCGGTCT (reverse).

**Western blotting**

Tissue samples were lysed in RIPA Lysis Buffer (Beyotime, P0013B) with protease and phosphatase inhibitors (Applygen, All-in-One, 100x), and 30 μg of protein was separated by SDS-PAGE. Proteins were transferred to a polyvinylidene fluoride membrane (Millipore, 0.45um) at 300 mA constant current for 90 minutes. For detection, membranes were incubated with primary antibodies overnight, then with horseradish peroxidase labeled secondary antibodies at room temperature. Then they were developed by a chemiluminescence imager. Western blot antibodies: SRF(1:1000, Cell Signaling Technology, 5147); p38 MAPK(1:1000, Cell Signaling Technology, 8690); MRTFA(1:1000, Cell Signaling Technology, 14760); beta-actin(1:2000, Proteintech, 20536-1-AP).

**References**

1. He L, Huang Y, Zhu H, Pang G, Zheng W, Wong Y-S, et al. Cancer-targeted monodisperse mesoporous silica nanoparticles as carrier of ruthenium polypyridyl complexes to enhance theranostic effects. *Advanced Functional Materials*. 2014;24:2754-2763

2. Williams RO. Collagen-induced arthritis as a model for rheumatoid arthritis. *Tumor necrosis factor*. Springer; 2004:207-216.

3. Trentham DE, Townes AS, Kang AH. Autoimmunity to type ii collagen an experimental model of arthritis. *The Journal of experimental medicine*. 1977;146:857-868

4. Brand DD, Latham KA, Rosloniec EF. Collagen-induced arthritis. *Nature protocols*. 2007;2:1269

5. O’Connell JX. Pathology of the synovium. *American journal of clinical pathology*. 2000;114:773-784

6. Naz S, Vallejo M, García A, Barbas C. Method validation strategies involved in non-targeted metabolomics. *Journal of Chromatography A*. 2014;1353:99-105

7. Chen J, Zhang X, Cao R, Lu X, Zhao S, Fekete A, et al. Serum 27-nor-5β-cholestane-3, 7, 12, 24, 25 pentol glucuronide discovered by metabolomics as potential diagnostic biomarker for epithelium ovarian cancer. *Journal of proteome research*. 2011;10:2625-2632

8. Yin P, Zhao X, Li Q, Wang J, Li J, Xu G. Metabonomics study of intestinal fistulas based on ultraperformance liquid chromatography coupled with q-tof mass spectrometry (uplc/q-tof ms). *Journal of proteome research*. 2006;5:2135-2143
