# Supplementary material for: A folate/RGD‐dual‐functionalized mesoporous silica nanoparticles targeting GABA‐p38 MAPK‐MRTFs/SRF signaling pathway in rheumatoid arthritis
Source: Clin Transl Med. 2021 May 7;11(5):e408. doi: 10.1002/ctm2.408 (PMC8103722; doi:10.1002/ctm2.408)
Supplement: Supplementary file 2 — Figure S1. (A and B) TEM images of MSNs, and it was observed that MSNs are spherical nanoparticles with a diameter of about 80 nm. Figure S2. Hydrodynamic diameter (A) and Zeta potential (B) of MSNs. The average hydrodynamic size of MSNs is 277.5 nm and their surface charge is 28.9 mV. Figure S3. SEM (A) and TEM (B) images of MSNs. SEM (C) and TEM (D) images of MSN‐NH2. No significant influence of ‐NH2 modification on the spherical morphology, size, as well as mesoporous property of MSNs was observed. Figure S4. Zeta potential of MSN‐NH2. The surface charge is about 16.9 mV. Figure S5. The FTIR spectra of MSNs and MSN‐NH2. A new FTIR spectra band appeared at 3421 cm‐1 after modification due to the stretching vibration of ‐NH2 groups. Figure S6. The linear relationship between the concentration of PD and their optical absorption intensity. The fitted linear equation is y = 0.04009 + 0.02998x, R2 = 0.99897. Figure S7. (A) N2 adsorption‐desorption isotherms and (B) pore‐size distributions of MSNs. (C) N2 adsorption‐desorption isotherms and (D) pore‐size distributions of PD@MSNs. Figure S8. (A) UV‐Vis spectra of PD@MSN‐NH2, NHS‐PEG‐FA, PD@MSN‐FA/SH. (B) Zeta potential of PD@MSN‐NH2, PD@NHS‐FA/SH, PD@MSN‐FA/RGD. Figure S9. H&E staining of pathological changes in response to different dosages of PD@MSN‐FA/RGD after 14 days’ administration. Figure S10. UPLC‐Q exactive quantitative analysis of polydatin in plasma, heart, liver, spleen, kidney, synovial fluid, and synovial membrane tissue of rats which subject to PLN injection based on selective ion monitoring (SIM)‐based method. Figure S11. Metabolic trait of polydatin in vivo. 7 major metabolites generated from polydatin degradation were detected in the liver of rats which subject to polydatin injection, while none significant degradation product was detected upon PD@MSN‐FA/RGD injection. Figure S12. Multi‐dimensional statistics of metabolomics data. A–B. PCA analysis based on LC‐MS data obtained from synovial fluid of Con [file CTM2-11-e408-s002.docx]

**Supplemental figures/tables**


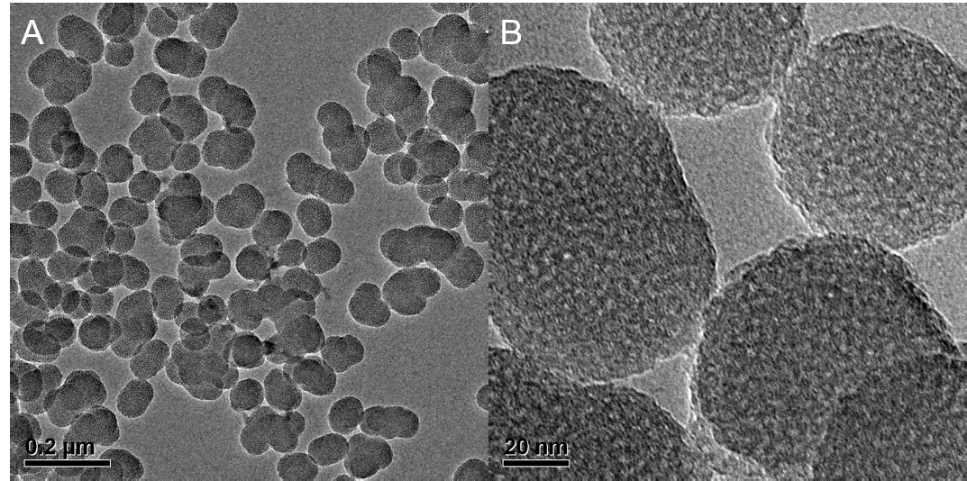


**Figure S1. A-B.** TEM images of MSNs, it was observed that MSNs are spherical nanoparticles with a diameter of about 80 nm.

**Figure S2.** Hydrodynamic diameter **(A)** and Zeta potential **(B)** of MSNs. Average hydrodynamic size of MSNs is 277.5 nm and their surface charge is 28.9 mV.


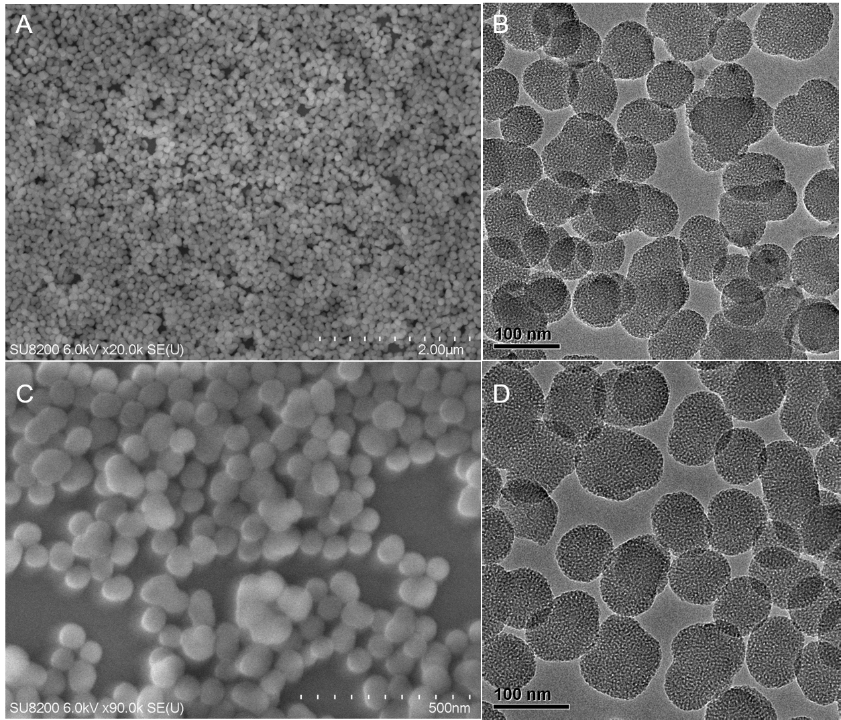


**Figure S3.** SEM **(A)** and TEM **(B)** images of MSNs. SEM **(C)** and TEM **(D)** images of MSN-NH_2_. No significant influence of -NH_2_ modification on the spherical morphology, size, as well as the mesoporous property of MSNs was observed.


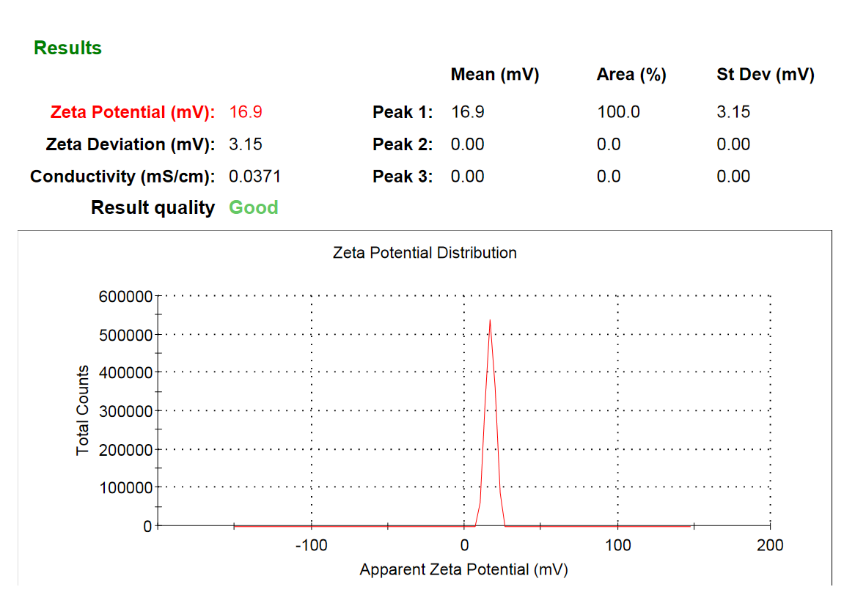


**Figure S4.** Zeta potential of MSN-NH_2_. The surface charge is about 16.9 mV.


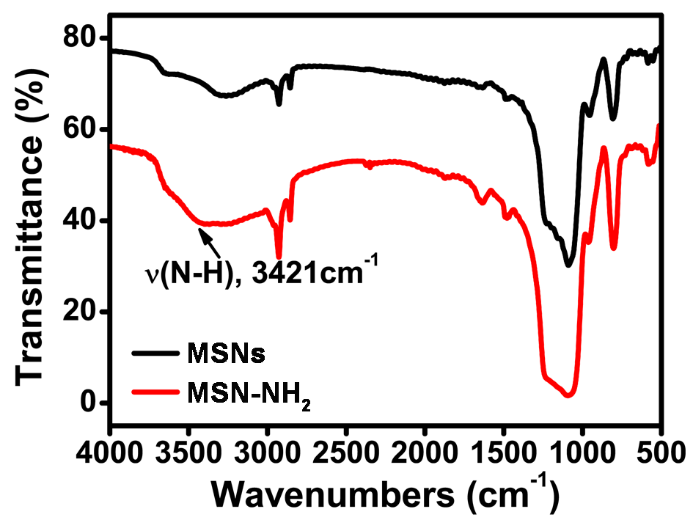


**Figure S5.** The FTIR spectra of MSNs and MSN-NH_2_. A new FTIR spectra band appeared at 3421 cm-1 after modification due to the stretching vibration of -NH_2_ groups.

**Figure S6.** The linear relationship between the concentration of PD and their optical absorption intensity. The fitted linear equation is y = 0.04009 + 0.02998x, R^2^ = 0.99897.


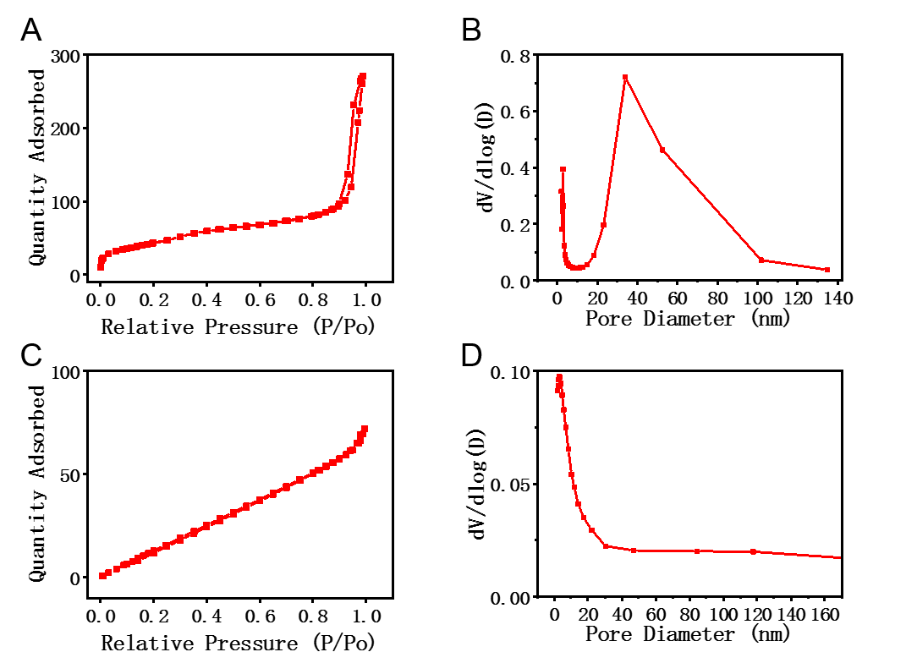


**Figure S7.** (A) N_2_ adsorption-desorption isotherms and (B) pore-size distributions of MSNs. (C) N_2_ adsorption-desorption isotherms and (D) pore-size distributions of PD@MSNs.


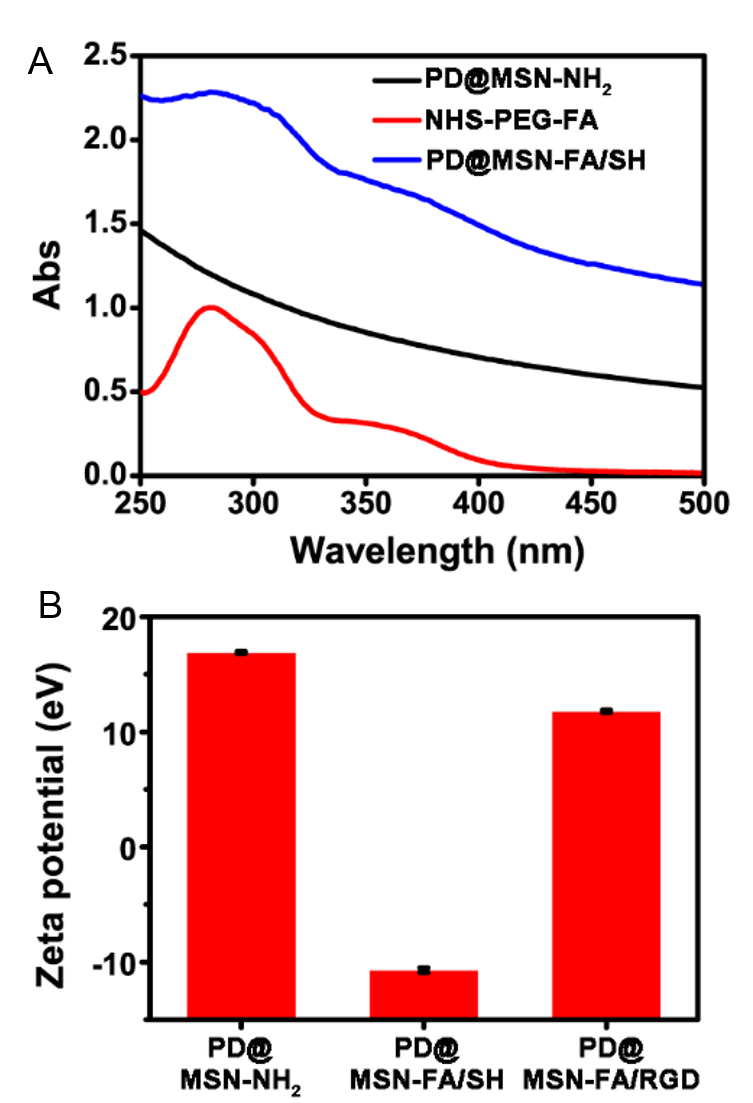


**Figure S8.** (A) UV-Vis spectra of PD@MSN-NH_2_, NHS-PEG-FA, PD@MSN-FA/SH. (B) Zeta potential of PD@MSN-NH_2_, PD@NHS-FA/SH, PD@MSN-FA/RGD.

**Figure S9.** H&E staining of pathological changes in response to different dosages of PD@MSN-FA/RGD after 14 days’ administration.


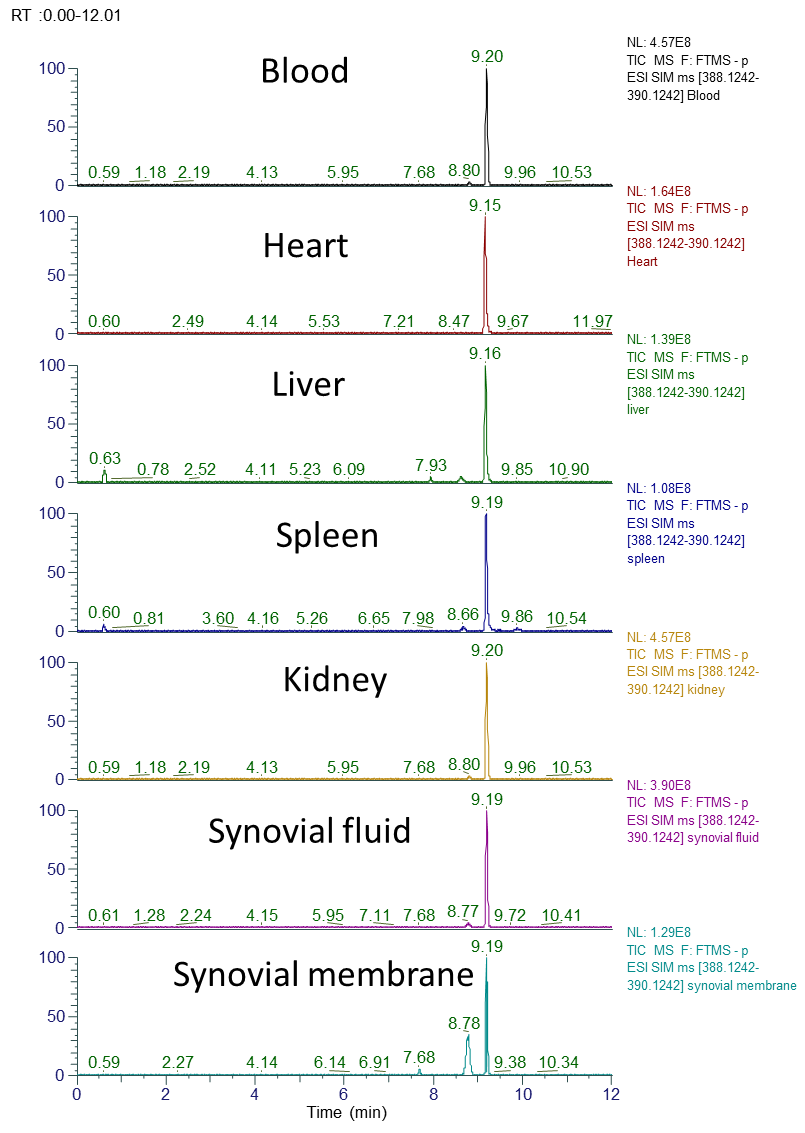


**Figure S10.** UPLC-Q Exactive quantitative analysis of polydatin in plasma, heart, liver, spleen, kidney, synovial fluid and synovial membrane tissue of rats which subject to PLN injection based on selective ion monitoring (SIM)-based method.

**Figure S11.** Metabolic trait of polydatin in vivo. 7 major metabolites which generate from polydatin degradation were detected in liver of rats which subject to polydatin injection, while none significant degradation product was detected upon PD@MSN-FA/RGD injection.

**Figure S12.** Multi-dimensional statistics of metabolomics data. A-B. PCA analysis based on LC-MS data obtained from synovial fluid of Control, Model, Positive, high-dose PLN, and low-dose PLN group. **(A)** Result based on HILIC mode data; **(B)** Result based on RP-C18 mode data; C-H. OPLS-DA on LC-MS data of common metabolites of control group, model group, positive group, high-dose PLN group, and low-dose PLN group. **(C, F)** Control vs. Model groups under HILIC and RP-C_18_ mode. (**D, G)** Model vs. high-dose PLN groups under HILIC and RP-C_18_ mode **(E, H)** Model vs. low-dose PLN groups under HILIC and RP-C_18_ mode; I-N. Robustness assessments of OPLS-DA model.

**Figure S13.** Hierarchical clustering analysis on common differential metabolites detected and identified in each group.

**Table S1.** The loading content and entrapment efficiency of PD@MSN and PD@MSN-NH_2_.

| MSN-NH_2_/PD  mass ratio | PD@MSN | |  | PD@MSN-NH_2_ | |
| --- | --- | --- | --- | --- | --- |
|  | Loading content (%) | Entrapment efficiency (%) |  | Loading content (%) | Entrapment efficiency (%) |
| 5 | 19.0 ± 0.3 | 95.1 ± 1.4 |  | 18.8 ± 0.5 | 94.1 ± 2.5 |
| 2 | 47.5 ± 0.4 | 95.1 ± 0.7 |  | 47.1 ± 1.5 | 94.2 ± 2.9 |
| 1 | 94.2 ± 1.7 | 94.2 ± 1.7 |  | 93.8 ± 3.3 | 93.8 ± 3.3 |

Data is presented as mean ± SD, t-test, PD@MSN *vs.* PD@MSN-NH_2_: *p <0.05, **p <0.01.

The loading content (LC) and entrapment efficiency (EE) can be calculated following the equations below

$$LC\left( \% \right)=\frac{M_{iD}-M_{sD}}{M}\times100\%$$

$$EE\left( \% \right)=\frac{M_{iD}-M_{sD}}{M_{iD}}$$

Where *M_iD_* is the initial amount of PD used, *M_SD_* is the amount of PD in the supernatant, M is the amount of MSN-NH_2_ used plus the amount of PD loaded into MSN-NH_2_.

**Table S2.** OD values of serum in mice subjecting to different dosage of PD@MSN-FA/RGD.

| Dosage | OD 450 nm | OD 540 nm |
| --- | --- | --- |
| 0mg/kg (control) | 0.43±0.05 | 0.27±0.04 |
| 54.78mg/kg | 0.37±0.06 | 0.21±0.05 |
| 109.57mg/kg | 0.35±0.02 | 0.21±0.02 |
| 219.145mg/kg | 0.41±0.07 | 0.24±0.05 |
| 438.28 mg/kg | 0.37±0.06 | 0.21±0.02 |

Data is presented as mean ± SD, t-test, compared within same measurement batch; *vs*. 0mg/kg: *p <0.05, **p <0.01.

**Table S3.** Organ coefficients of heart, liver, spleen, lung, and kidney in mice subjecting to different dosage of PD@MSN-FA/RGD.

| Group | Heart | Liver | Spleen | Lung | Kidney |
| --- | --- | --- | --- | --- | --- |
| 0mg/kg (control) | 0.70±0.08 | 4.70±0.28 | 0.38±0.06 | 0.77±0.16 | 1.75±0.16 |
| 54.78mg/kg | 0.58±0.03 | 4.76±0.16 | 0.37±0.04 | 0.75±0.01 | 1.81±0.20 |
| 109.57mg/kg | 0.65±0.10 | 4.84±0.28 | 0.38±0.03 | 0.75±0.05 | 1.75±0.11 |
| 219.145mg/kg | 0.58±0.04 | 4.68±0.15 | 0.39±0.03 | 0.77±0.03 | 1.75±0.07 |
| 438.28 mg/kg | 0.63±0.04 | 4.95±0.24 | 0.45±0.02 | 0.72±0.08 | 1.56±0.08 |

Data is presented as mean ± SD, t-test, compared within same measurement batch; *vs.* control group: *p <0.05, **p <0.01.

**Table S4.** Horizontal diameters of right hind legs (mm)

| Group | N | 0^th^week | 1^st^week | 2^nd^ week | 3^rd^ week | 4^th^ week |
| --- | --- | --- | --- | --- | --- | --- |
| Control | 6 | 6.38±0.21 | 6.41±0.27 | 6.09±0.27 | 6.88±0.37 | 7.02±0.11 |
| Model | 6 | 8.23±0.53** | 8.29±1.03** | 10.59±1.49** | 10.57±0.75** | 9.5±0.81** |
| Positive | 8 | 8.52±0.58** | 8.86±0.74 | 9.69±1.23 | 11.04±1.32 | 9.43±1.11 |
| High-dose | 7 | 8.85±0.87** | 8.85±0.87 | 9.07±1.1# | 10.14±0.57 | 9.15±0.74 |
| Low-dose | 7 | 8.55±0.83** | 8.54±0.83 | 9.18±1.76 | 10.21±1.49 | 9.13±1.15 |

Data is presented as mean ± *SD*, *t-test*, compared within same measurement batch; *vs.* control group: *p <0.05, **p <0.01; *vs.* model group: #p <0.05, ##p <0.01.

**Table S5.** Vertical diameters of right hind legs(mm)

| Group | N | 0^th^week | 1^st^week | 2^nd^ week | 3^rd^ week | 4^th^ week |
| --- | --- | --- | --- | --- | --- | --- |
| Control | 6 | 5.17±0.17 | 5.17±0.17 | 5.06±0.4 | 4.94±0.35 | 5.61±0.41 |
| Model | 6 | 9.3±2.55** | 10.95±2.44** | 11.45±1.24** | 11.75±1.05** | 11.49±1.6** |
| Positive | 8 | 10.91±1** | 11.49±1.55 | 10.84±1.27 | 11.05±1.54 | 11.45±1.58 |
| High-dose | 7 | 11.17±1.41** | 11.50±1.58 | 9.69±0.99## | 10.85±0.99 | 10.22±1.24 |
| Low-dose | 7 | 10.84±1.39** | 11.82±1.71 | 10.53±0.91 | 10.6±1.18 | 10.1±0.77 |

Data is presented as mean ± *SD*, *t-test*, compared within same measurement batch; *vs.* control group: *p <0.05, **p <0.01; *vs.* model group: #p <0.05, ##p <0.01.

**Table S6.** Horizontal diameters of left hind legs(mm)

| Group | N | 0^th^week | 1^st^week | 2^nd^ week | 3^rd^ week | 4^th^ week |
| --- | --- | --- | --- | --- | --- | --- |
| Control | 6 | 6.24±0.33 | 6.05±0.28 | 6.21±0.3 | 6.56±0.38 | 6.91±0.19 |
| Model | 6 | 8.34±0.76** | 8.7±0.62** | 10.25±2.29** | 10.12±0.16** | 9.93±0.2* |
| Positive | 8 | 8.14±1.23** | 8.92±0.79 | 9.9±1.36 | 9.75±1.3 | 8.94±0.8# |
| High-dose | 7 | 7.89±0.55** | 8.82±1.01 | 9.3±1.57 | 10.03±1.2 | 9.2±0.13 |
| Low-dose | 7 | 7.89±0.9** | 9.02±0.95 | 9.34±1.54 | 9.22±0.94 | 8.44±0.92# |

Data is presented as mean ± *SD*, *t-test*, compared within the same measurement batch; *vs.* control group: *p <0.05, **p <0.01; *vs.* model group: #p <0.05, ##p <0.01.

**Table S7.** Vertical diameters of left hind legs(mm)

| Group | N | 0^th^week | 1^st^week | 2^nd^ week | 3^rd^ week | 4^th^ week |
| --- | --- | --- | --- | --- | --- | --- |
| Control | 6 | 4.97±0.25 | 4.98±0.21 | 5.08±0.22 | 5.19±0.19 | 6.91±0.19 |
| Model | 6 | 11.14±1.24** | 12.64±1.41** | 11.26±1.41** | 12.38±0.77** | 9.93±0.2* |
| Positive | 8 | 10.31±1.2** | 11.18±1.34# | 11.05±1.26 | 11.59±1.17 | 8.94±0.8# |
| High-dose | 7 | 10.33±1.1** | 10.49±0.94# | 9.82±1.01# | 11.03±1.99 | 9.2±0.13 |
| Low-dose | 7 | 10.7±1.14** | 11.37±1.2 | 9.85±0.68# | 10.56±1.28# | 8.44±0.92# |

Data is presented as mean ± *SD*, *t-test*, compared within same measurement batch, *vs.* control group: *p <0.05, **p <0.01; *vs.* model group: #p <0.05, ##p <0.01.

**Table S8.** Thickness of right hind foot pads(mm)

| Group | N | 0^th^week | 1^st^week | 2^nd^ week | 3^rd^ week | 4^th^ week |
| --- | --- | --- | --- | --- | --- | --- |
| Control | 6 | 3.26±0.23 | 3.14±0.18 | 3.46±0.17 | 3.28±0.68 | 3.79±0.72 |
| Model | 6 | 5.13±0.27** | 5.67±0.39** | 5.66±0.34** | 6.41±0.39** | 6.29±0.51** |
| Positive | 8 | 5.19±0.89** | 5.59±0.53 | 5.16±0.81 | 6.7±0.64 | 6.08±0.8 |
| High-dose | 7 | 5.35±0.93** | 5.26±0.86 | 5.15±0.82 | 6.2±0.37 | 5.66±0.65 |
| Low-dose | 7 | 5.25±0.4** | 5.88±0.33 | 4.84±0.39# | 5.98±0.29 | 5.23±0.36# |

Data is presented as mean ± *SD*, *t-test*, compared within same measurement batch; *vs.* control group: *p <0.05, **p <0.01; *vs.* model group: #p <0.05, ##p <0.01.

**Table S9.** Thickness of left hind foot pads(mm)

| Group | N | 0^th^week | 1^st^week | 2^nd^ week | 3^rd^ week | 4^th^ week |
| --- | --- | --- | --- | --- | --- | --- |
| Control | 6 | 3.13±0.27 | 3.23±0.3 | 3.41±0.15 | 3.25±0.36 | 3.78±0.36 |
| Model | 6 | 5.16±1.49** | 5.27±1.24** | 5.9±0.3** | 6.31±0.41** | 6.05±0.49** |
| Positive | 8 | 4.51±0.76** | 5.47±0.78 | 5.78±0.39 | 6.59±0.75 | 5.97±0.37 |
| High-dose | 7 | 4.69±0.61** | 5.3±0.42 | 5.14±0.55# | 5.97±0.33 | 5.71±0.42 |
| Low-dose | 7 | 4.74±0.83** | 5.37±0.88 | 5.51±0.61 | 6.14±0.34 | 5.84±0.47 |

Data is presented as mean ± *SD*, *t-test*, compared within same measurement batch, *vs.* control group: *p <0.05, **p <0.01; *vs.* model group: #p <0.05, ##p <0.01.

**Table S10.** Vertical and horizontal diameters of left/right ankle joints

| Group | N | Right- horizontal | Right-vertical | Left- horizontal | Left- vertical |
| --- | --- | --- | --- | --- | --- |
| Control | 6 | 6.295±0.238 | 7.843±0.411 | 5.911±0.279 | 7.003±0.722 |
| Model | 6 | 7.59±0.533** | 8.565±1.227 | 8.3±0.429** | 10.143±0.483** |
| Positive | 8 | 7.351±0.657 | 8.406±0.832 | 7.48±0.702# | 9.238±0.659## |
| High-dose | 7 | 7.149±0.861 | 7.776±0.79 | 7.549±0.846# | 7.996±0.65## |
| Low-dose | 7 | 7.514±0.673 | 7.924±1.44 | 7.464±0.502# | 7.064±0.921## |

Data is presented as mean ± *SD*, *t-test*, compared within same measurement batch, *vs.* control group: *p <0.05, **p <0.01; *vs.* model group: #p <0.05, ##p <0.01.

**Table S11.** Differential metabolites being detected and identified.

| ID | metabolites | platform | tR/min | Formula | Weight | Ratio(model/ control | Ratio:(positive/ model | Ratio(high / model) | Ratio(low/model) | P-value(model/control) | P-value(positive/model) | P-value(high )/model) | P-value(low / model) |
| --- | --- | --- | --- | --- | --- | --- | --- | --- | --- | --- | --- | --- | --- |
| 1 | 1-oleoyl-GPE (18:1) | HILIC | 1.595 | C_23_H_46_NO_7_P | 479.30107 | 1.258 | 0.057 | 0.409 | 0.568 | 0.97742903 | 8.2102E-06 | 0.31406069 | 0.91710151 |
| 2 | 5-Methylcytosine | HILIC | 5.175 | C_5_H_7_N_3_O | 125.05882 | 1.064 | 0.466 | 0.422 | 0.199 | 0.99983045 | 0.15197471 | 0.45675661 | 0.02746715 |
| 3 | Acetyl-L-carnitine | HILIC | 1.598 | C_9_H_17_NO_4_ | 203.11538 | 1.164 | 0.047 | 0.653 | 0.402 | 0.79091331 | 0.00306579 | 0.85234641 | 0.45295379 |
| 4 | L-arginine | HILIC | 11.008 | C_6_H_14_N_4_O_2_ | 174.11127 | 2.144 | 0.028 | 0.323 | 0.617 | 0.32617121 | 0.00015017 | 0.08716065 | 0.98602758 |
| 5 | benzoate | HILIC | 5.461 | C_7_H_6_O_2_ | 122.03668 | 10.595 | 0.322 | 0.348 | 0.346 | 0.03270355 | 0.42065015 | 0.42107867 | 0.77884368 |
| 6 | butyrylcarnitine | HILIC | 1.57 | C_11_H_21_NO_4_ | 231.14655 | 1.756 | 0.014 | 0.686 | 0.619 | 0.99750491 | 0.03772987 | 0.99626578 | 0.91074774 |
| 7 | decanoylcarnitine (C10) | HILIC | 1.532 | C_17_H_33_NO_4_ | 315.24003 | 1.679 | 0.088 | 0.423 | 0.525 | 0.27680353 | 2.09874E-0 | 0.74691581 | 0.30080514 |
| 8 | D-Serine | HILIC | 8.332 | C_3_H_7_NO_3_ | 105.04277 | 105.417 | 0.456 | 0.006 | 0.007 | 0.00082935 | 0.6755286 | 8.05563E-0 | 7.30348E-0 |
| 9 | guanidinoacetate | HILIC | 5.482 | C_3_H_7_N_3_O_2_ | 117.05386 | 11.93 | 0.085 | 0.111 | 0.105 | 0.04841436 | 0.02872547 | 0.32132722 | 0.25940833 |
| 10 | guanine | HILIC | 6.318 | C_5_H_5_N_5_O | 151.04914 | 3.129 | 0.267 | 0.165 | 0.303 | 0.21031855 | 0.09802778 | 0.02257244 | 0.14261589 |
| 11 | HMBA | HILIC | 1.579 | C_10_H_20_N_2_O_2_ | 200.15209 | 2.082 | 0.045 | 0.481 | 0.488 | 0.28736235 | 3.99031E-0 | 0.82046587 | 0.28489588 |
| 12 | indoleacetate | HILIC | 1.523 | C_10_H_9_NO_2_ | 175.06293 | 1.137 | 0.076 | 0.76 | 0.648 | 0.94412640 | 0.00567167 | 0.99268818 | 0.82719819 |
| 13 | isoleucylisoleucine | HILIC | 1.574 | C_12_H_24_N_2_O_3_ | 244.17814 | 1.909 | 0.105 | 0.185 | 0.203 | 0.95172731 | 0.00742046 | 0.37714325 | 0.54650845 |
| 14 | kynurenic acid | HILIC | 1.494 | C_10_H_7_NO_3_ | 189.04223 | 1.982 | 0.112 | 0.711 | 0.483 | 0.42508100 | 0.02030402 | 0.75857223 | 0.58332124 |
| 15 | Leu-pro | HILIC | 1.681 | C_11_H_20_N_2_O_3_ | 228.14686 | 1.81 | 0.099 | 0.476 | 0.366 | 0.39142837 | 0.00163629 | 0.51772645 | 0.52663400 |
| 16 | L-Histidine | HILIC | 11.415 | C_6_H_9_N_3_O_2_ | 155.06928 | 1.701 | 0.094 | 0.487 | 1.323 | 0.82360710 | 0.00144315 | 0.89193751 | 0.71755140 |
| 17 | N-acetylleucine | HILIC | 1.58 | C_8_H_15_NO_3_ | 173.10485 | 5.469 | 0.043 | 0.445 | 0.567 | 0.07372533 | 0.00044953 | 0.70372401 | 0.85841798 |
| 18 | N-acetylserine | HILIC | 7.752 | C_5_H_9_NO_4_ | 147.05286 | 13.55 | 0.49 | 0.551 | 0.423 | 0.02714960 | 0.66598313 | 0.92534796 | 0.19338371 |
| 19 | Phenethylamine | HILIC | 1.559 | C_8_H_11_N | 121.08907 | 0.503 | 1.725 | 1.547 | 1.197 | 0.04825763 | 0.19307531 | 0.16374388 | 0.35375445 |
| 20 | p-Xylene | HILIC | 1.58 | C_8_H_10_ | 106.07837 | 6.761 | 0.021 | 0.299 | 0.41 | 0.21034672 | 0.00036942 | 0.81637411 | 0.75732953 |
| 21 | tiglylcarnitine (C5:1-DC) | HILIC | 1.568 | C_12_H_21_NO_4_ | 243.14672 | 2.87 | 0.126 | 0.4 | 0.439 | 0.43657304 | 0.02285153 | 0.92953867 | 0.57452419 |
| 22 | Acetanilide | RP-C_18_ | 0.984 | C_8_H_9_NO | 135.0681 | 2.408 | 0.862 | 0.353 | 0.238 | 0.10188069 | 0.99676031 | 0.01606017 | 0.00143754 |
| 23 | Choline | RP-C_18_ | 0.871 | C_5_H_13_NO | 103.09942 | 1.29 | 0.88 | 0.696 | 0.41 | 0.94503446 | 0.96837023 | 0.87143845 | 0.01294352 |
| 24 | Cytidine | RP-C_18_ | 0.876 | C_9_H_13_N_3_O_5_ | 265.06688 | 6.25 | 0.952 | 0.198 | 0.118 | 0.14314621 | 0.99725111 | 0.12045080 | 0.01505379 |
| 25 | eicosapentaenoate (EPA; 20:5n3) | RP-C_18_ | 10.257 | C_20_H_30_O_2_ | 302.22402 | 0.369 | 3.597 | 0.665 | 0.286 | 0.99495434 | 0.6979005 | 0.34238980 | 0.02942826 |
| 26 | Hypoxanthine | RP-C_18_ | 1.021 | C_5_H_4_N_4_O | 136.03822 | 1.691 | 0.466 | 0.159 | 0.114 | 0.41011731 | 0.30442273 | 0.02220087 | 0.00292778 |
| 27 | phenylpyruvate | RP-C_18_ | 0.984 | C_9_H_8_O_3_ | 164.04703 | 2.706 | 0.971 | 0.401 | 0.282 | 0.10587752 | 0.99999825 | 0.04845405 | 0.00551490 |
| 28 | p-Xylene | RP-C_18_ | 1.511 | C_8_H_10_ | 106.07799 | 7.858 | 0.8 | 0.262 | 0.244 | 0.04657179 | 0.9610596 | 0.29390726 | 0.30388387 |
